# Psychological factors and DNA methylation of genes related to immune/inflammatory system markers: the VA Normative Aging Study

**DOI:** 10.1136/bmjopen-2015-009790

**Published:** 2016-01-05

**Authors:** Daniel Kim, Laura D Kubzansky, Andrea Baccarelli, David Sparrow, Avron Spiro, Letizia Tarantini, Laura Cantone, Pantel Vokonas, Joel Schwartz

**Affiliations:** 1 Department of Health Sciences, Northeastern University, Boston, Massachusetts, USA; 2 Department of Social and Behavioral Sciences, Harvard School of Public Health, Boston, Massachusetts, USA; 3 Department of Human and Social Sciences, EHESP French School of Public Health, Rennes, France; 4 Department of Environmental Health, Harvard School of Public Health, Boston, Massachusetts, USA; 5 Boston University School of Medicine, Boston, Massachusetts, USA; 6 VA Normative Aging Study, VA Boston Healthcare System, Boston, Massachusetts, USA; 7 Department of Epidemiology, Boston University School of Public Health, Boston, Massachusetts, USA; 8 Department of Psychiatry, Boston University School of Medicine, Boston, Massachusetts, USA; 9 University of Milan, Milan, Italy; 10 IRCCS Maggiore Policlinico Hospital, Milan, Italy; 11 Department of Epidemiology, Harvard School of Public Health, Boston, Massachusetts, USA

**Keywords:** psycholoogical factors, methylation

## Abstract

**Objectives:**

Although psychological factors have been associated with chronic diseases such as coronary heart disease (CHD), the underlying pathways for these associations have yet to be elucidated. DNA methylation has been posited as a mechanism linking psychological factors to CHD risk. In a cohort of community-dwelling elderly men, we explored the associations between positive and negative psychological factors with DNA methylation in promoter regions of multiple genes involved in immune/inflammatory processes related to atherosclerosis.

**Design:**

Prospective cohort study.

**Setting:**

Greater Boston, Massachusetts area.

**Participants:**

Samples of 538 to 669 men participating in the Normative Aging Study cohort with psychological measures and DNA methylation measures, collected on 1–4 visits between 1999 and 2006 (mean age=72.7 years at first visit).

**Outcome measures:**

We examined anxiety, depression, hostility and life satisfaction as predictors of leucocyte gene-specific DNA methylation. We estimated repeated measures linear mixed models, controlling for age, smoking, education, history of heart disease, stroke or diabetes, % lymphocytes, % monocytes and plasma folate.

**Results:**

Psychological distress measured by anxiety, depression and hostility was positively associated, and happiness and life satisfaction were inversely associated with average Intercellular Adhesion Molecule-1 (*ICAM-1*) and coagulation factor III (*F3*) promoter methylation levels. There was some evidence that hostility was positively associated with toll-like receptor 2 (*TLR-2*) promoter methylation, and that life satisfaction was inversely associated with *TLR-2* and inducible nitric oxide synthase (*iNOS*) promoter methylation. We observed less consistent and significant associations between psychological factors and average methylation for promoters of the genes for glucocorticoid receptor (*NR3C1*), interferon-γ (*IFN-γ*) and interleukin 6 (*IL-6*).

**Conclusions:**

These findings suggest that positive and negative psychological factors affect DNA methylation of selected genes involved in chronic immune/inflammatory processes and inflammation-related endothelial dysfunction. Such epigenetic changes may represent biological pathways that mediate the effects of psychological factors on CHD.

**Strengths and limitations of this study**

- Strengths of our study include its novel examination of multiple psychological factors (both positive and negative) in relation to DNA methylation in promoter regions of multiple genes plausibly involved in chronic immune/inflammatory processes and inflammation-related endothelial dysfunction.
- We also used repeated measures, thereby improving precision of our estimates.
- A subset of CpG sites was examined for DNA methylation within a gene promoter region; the CpGs from these sites may not necessarily have been good proxies for all CpGs within the same region.
- The study sample was limited to an elderly, primarily white male population and associations of psychological factors with DNA methylation may be more salient in other population subgroups.

## Introduction

Although psychological factors and clinical disorders such as anxiety and depression have been linked to a wide variety of health and disease end points including coronary heart disease (CHD) in epidemiological studies,^1–3^ the mechanisms that underlie the associations with CHD have yet to be fully elucidated. CHD has been increasingly characterised as a chronic inflammatory process involving such factors as intercellular adhesion molecules (ie, Intercellular Adhesion Molecule-1 (*ICAM-1*), Vascular Cell Adhesion Molecule-1 (*VCAM-1*)) facilitating the transendothelial migration of inflammation-related cells into vascular tissues.^4^

DNA methylation may be an intermediary mechanism by which psychological factors influence CHD risk. DNA methylation is a reversible process corresponding to the addition of methyl groups at the 5’ position of cytosine rings in CpG dinucleotides to produce 5-methyl-cytosine (5mC). DNA methylation is involved in regulation of gene expression and in several genes, lower methylation has been associated with increased mRNA expression.^5^ These relatively stable epigenetic marks can modify gene expression for proteins shaping cellular signals, responses and function. Such modifications may underlie the pathogenesis of major chronic diseases including CHD and cancer.^6–8^ In humans, lower levels of blood LINE-1 DNA methylation have predicted higher risks of cardiovascular diseases,^9^ and alterations in the DNA methylation of specific genes have been linked to higher risks of CHD and cancer.^10^ ^11^

Recent experimental and epidemiological evidence suggests that social/psychological exposures may contribute to the methylation of selected genes/promoters, and may thereby influence gene expression relevant to disease risk factors.^5^ ^12–17^ In rats, Weaver *et al*^5^ found that low levels of maternal licking and grooming led to *higher* cytosine methylation in a glucocorticoid receptor (*NR3C1*) promoter region in the brain hippocampus of offspring. Such hypermethylation is linked to lower GR expression. Since *NR3C1* up-regulation induces negative feedback in the hypothalamic-pituitary-adrenal (HPA) axis,^18^ ^19^ its hypothesised downregulation with negative psychological exposures would potentially generate proinflammatory stress responses. In humans, one study has reported associations between higher anxiety and depressive symptom scores in prenatal women and higher methylation of the *NR3C1* gene in newborn cord blood leucocytes and maternal blood leucocytes.^12^ A study of younger to middle-aged adults found correlations between a history of childhood adversity with higher leucocyte *NR3C1* gene promoter methylation, although no correlations for anxiety and limited correlations for depression with *NR3C1* promoter methylation were found.^20^ Distinct methylation patterns have been further observed in depressed versus not depressed individuals,^13^ and lower job seniority has been linked to higher global (*Alu* line) methylation and methylation in interferon-γ (IFN-γ) promoter regions.^14^ Furthermore, individuals of low socioeconomic status (SES) in early life with mothers who expressed high warmth toward them were shown to exhibit less Toll-like receptor (TLR)-stimulated production of interleukin-6 (IL-6)^21^; IL-6 is an inflammatory marker that is predicted by psychosocial factors such as anxiety and depression, and is thought to be involved in the pathogenesis of cardiovascular disease.^22^ Overall, these studies suggest that aspects of the social environment and mood disorders, including anxiety and depression, may induce epigenetic effects.^23^ ^24^ Plausibly, these epigenetic changes represent underlying common biological (eg, immune, neuroendocrine) pathways for the putative effects of psychological factors on chronic diseases including CHD.

In a cohort of community-dwelling elderly men in the USA, we explored the associations between positive and negative psychological factors, and DNA methylation in promoter regions of multiple genes involved in chronic immune/inflammatory processes and inflammation-related endothelial dysfunction. These genes include those for the proteins noted above and for *F3* (also known as Tissue Factor) and *iNOS*, which have been shown to be involved in chronic inflammatory pathways and have been previously linked to chronic inflammatory conditions.^25–30^

To the best of our knowledge, this is the first study to examine a comprehensive set of psychological factors in relation to epigenetic processes plausibly related to CHD.

## Materials and methods

### Study population

The Normative Aging Study (NAS) is a longitudinal study of ageing established by the US Veterans Administration. The original cohort was recruited between 1961 and 1970, and consisted of 2280 community-dwelling men, aged 21–80 years, from the greater Boston, Massachusetts area, who were free of known chronic medical conditions at enrolment.^31^ Study participants have, every 3 to 5 years, undergone routine physical examinations and laboratory tests, and responded to surveys on medical history, and lifestyle and psychological factors.

The present study analysed data on men participating in the NAS cohort, with psychological measures and DNA methylation measures (average of 2.2 measures/individual), collected on between one to four visits between 1999 and 2006. During this period, 765 study participants provided at least one whole blood sample that was used to measure DNA methylation. Since for some participants the extracted DNA was not sufficient in quantity to conduct methylation assays for all genes, and due to some assay failures, the total numbers of men in whom there were assays corresponding to promoter regions of different genes varied.^32^

### Outcome variables

The average and position-specific levels of methylation in promoter regions of seven genes (toll-like receptor 2 (*TLR-2*), coagulation factor III (*F3*), glucocorticoid receptor (*NR3C1*), intercellular adhesion molecule-1 (*ICAM-1*), interferon-γ (*IFN-γ*), interleukin 6 *(IL-6*) and inducible nitric oxide synthase (*iNOS*)) were analysed as outcomes in separate models.

These genes were selected based on past evidence for associations of: (1) proteins coded by these genes in animal and/or human studies of atherosclerosis or the pathophysiology of heart disease; (2) psychological factors with methylation of promoters of the genes; (3) psychological factors with peripheral blood levels of the markers expressed by these genes. For instance, for the first selection criterion, both serum *ICAM-1* and *IL-6* levels have independently predicted CHD risk in prospective studies after controlling for demographic/socioeconomic and traditional CHD risk factors.^33^ ^34^ In the Introduction, we cited studies suggesting linkages between psychological exposures and the methylation of *NR3C1* and *IFN-γ* promoters, which in turn might explain chronic inflammatory processes characterising diseases such as CHD. As an example for the third selection criterion, lower early-life socioeconomic status (SES) has been linked to greater expression of both *NR3C1* and *TLR* receptor mRNA in leucocytes.^35^

DNA was extracted from a stored frozen buffy coat of 7 mL whole blood, using the QiAmp DNA blood kits (QIAGEN, Hilden, Germany); 500 ng DNA (concentration 50 ng/μL) was treated using EZ DNA Methylation-Gold Kit (Zymo Research, Orange, California, USA) according to the manufacturer's protocol. Final elution was performed with 30 μL of M-Elution Buffer.

CpG dinucleotide-rich promoter regions were identified using the Genomatix Software Suite (Genomatix, Germany). Promoters without any assigned transcripts were excluded. To the best of our knowledge, there were no DNA methylation assays for the genes analysed that were already published. Therefore, we developed new pyrosequencing assays by selecting amplicons in promoter CpG-rich areas. For each gene, the PCR-pyrosequencing primer (more than 20 base pairs long) of the highest available quality that was associated with one of the promoters was designed using specialised software (PSQ Assay Design, Biotage, Sweden). The fractions of CpG sites examined by gene were as follows: *TLR-2* (5/49); *F3* (5/78); *NR3C1* (1/7); *ICAM-1* (5/69); *IFN-γ* (2/8); *IL-6* (2/18); *iNOS* (2/8). We did not assay higher proportions of CpG sites due to inherent limitations of the method applied, that is, we excluded PCR amplicons with 350 base pairs or longer, primers that avoided CpGs and target sequences of 40 base pairs or longer, to optimise PCR and sequencing conditions. Online supplementary table S1 lists the specific CpG positions for DNA methylation that we measured within specified promoter regions for each gene. We had limited information about the CpGs that were analysed (eg, for *NR3C1*), including their functionality or their proximity to transcription factor-binding sites or other important sequences. Since genomic locations were for the hg18 genome build, the majority of the CpGs that we examined were not assayed by the most common methylation assays (ie, either the 27 K or 450 K assays) that are available in public data sets.

The degree of methylation was calculated as the percentage of methylated cytosine residues divided by the sum of methylated and unmethylated cytosine residues (%5mC) in each sample. Built-in controls were used to verify bisulfite conversion efficiency. Each sample was tested twice for each marker to improve statistical power and precision. The average of the replicates was used.

### Predictor variables

We used data on anxiety and depression measured through the Brief Symptom Inventory (BSI), a self-administered 53-item questionnaire of nine primary psychological symptom dimensions (anxiety, depression, hostility, interpersonal sensitivity, obsessive-compulsive, paranoid ideation, phobic anxiety, psychoticism, somatisation) experienced by the respondent over the previous 30 days; the BSI was included as part of the Health and Social Behavior Survey in the NAS, starting in 1985.^31^ ^36^ Happiness (based on the single item ‘How happy are you right now?’) and life satisfaction (based on the 11-item version of the Life Satisfaction Inventory-A)^37^ were also examined as predictor variables. Higher life satisfaction scale scores corresponded to higher self-reported life satisfaction; higher scores on the other scales reflected higher negative psychological symptoms. All psychological measures were analysed as continuous. Internal consistency reliability (Cronbach's α) values for the anxiety, depression, hostility and life satisfaction scales were all acceptably high (>0.70).

### Covariates

Model covariates consisted of the age at first visit in or after 1999 (years), smoking (pack-years of smoking), education (>high school, ≤high school), history of CHD or stroke prior to 1999, history of diabetes prior to 1999, % basophils, % eosinophils, % lymphocytes, % monocytes, % neutrophils and plasma folate levels. Previous evidence suggests that leucocyte composition is related to DNA methylation,^38^ and that folate is a source of methyl groups and folate depletion leads to lower blood DNA methylation.^39^ Since 98% of the sample was white, we did not adjust for race/ethnicity. In sensitivity analyses, we additionally controlled for baseline hypertension (ie, hypertension prior to 1999) and total serum cholesterol.

### Statistical analysis

We first calculated descriptive statistics (mean, range, percentages for psychological factors and covariates, mean percentage methylation for gene-specific promoter methylation) based on study participants with measures of ICAM-1 promoter region methylation, which showed several significant associations.

We then constructed a Pearson correlation coefficient matrix for the psychological factors and a correlation coefficient matrix for the methylation outcomes.

To examine the associations between the psychological factors and the methylation outcomes, we next estimated repeated measures linear mixed models (equivalent to random intercept models) to account for up to four repeated measures, using a first-order autoregressive covariance structure (in which a decreasing correlation of SEs over time was modelled). The log-likelihood fit statistics for the models indicated better model fits than those for the corresponding models using a compound symmetry covariance structure; unstructured covariance structure models did not converge. Since we assumed a short latency period for methylation changes,^40–43^ we modelled each psychological factor as a predictor of gene-specific methylation measured on the same visit (averaged across cytosines in CpG sites within the promoter region, varying from one CpG site for the *NR3C1* gene to five CpG sites for the *F3* gene according to the density of CpG sites in the sequence amplified within the promoter region). In addition, we noted the associations between selected covariates (age, smoking, income/education) and methylation.

For primary associations significant at the 5% level, we further tested for dose–response relationships, by grouping the respective psychological factor into meaningful and/or equally-sized categories where possible. A dose–response relationship would lend support to a casual association.^44^ A linear test for trend was performed by converting the categories into an ordinal variable and noting its corresponding p value.

We further examined the associations between psychological factors and serum ICAM-1, to examine whether similar relationships were present as between the psychological factors and ICAM-1 promoter methylation levels (because the latter would be expected to be inversely related to ICAM-1 expression).

Finally, because of the known association between ageing and methylation, we repeated the analyses using age^2^ as an additional covariate to saturate the model for an age effect, and found comparable results (data not shown). Additional sensitivity analyses explored the robustness of the findings after controlling for household income, baseline hypertension and total serum cholesterol.

All tests were two-tailed with a 5% significance level. All analyses were conducted using SAS V.9.1 (SAS Institute, Cary, North Carolina, USA).

All participants gave written informed consent. This research was approved by the human subjects committees of the Boston VA Medical Center and the Harvard School of Public Health.

## Results

### Characteristics of study sample

Table 1 shows descriptive characteristics of the study sample based on 616 men with measures of ICAM-1 promoter region methylation. We present characteristics for this sample because several of the corresponding associations with ICAM-1 methylation were significant among the different gene promoter regions analysed. The sample had a mean age of 72.5 years (range 56–100 years) at first visit. Approximately one-third (34.1%) attained no more than high school education and over two-thirds had previously smoked, with an average of 21.8 pack-years of smoking (table 1). These characteristics were similar to those of the larger cohort of men with visits between 1999 and 2006, including men with missing observations for methylation (n=1121 men: mean age 71.7 years, % with less than high school education=35.9; mean pack-years of smoking=21.6). After listwise deletion of missing data in respective models, the sizes of analytic samples ranged from 481 to 669 men. Missing gene-specific methylation data ranged from 5.4% (*IFN-γ*) to 23.8% (*iNOS*), due to the presence of assay failures and the lack of sufficient DNA, which disproportionately affected genes that were tested later in the order (ie, *iNOS*, *ICAM-1*). Missing model covariate data ranged collectively from 3.1% to 3.5%. Missing psychological factor data ranged from 3.7% (happiness) to 10.8% (life satisfaction) in the respective model (see online supplementary table S2). Mean leucocyte methylation levels within promoter regions ranged from 2.2% 5mC (OGG gene) to 84.8% 5mC (IFN-γ gene); none of the distributions was highly skewed (table 1). Intraindividual changes in leucocyte methylation ranged from 1.4 to 2.4 times the SD across repeated measures.

**Table 1:** Descriptive statistics (mean values with ranges in parentheses; percentages) for samples analysed with respective characteristic and ICAM-1 promoter methylation (n ranging from 538 to 577 men)

Mean age in years at first visit in 1999	72.5 (56–100)
Percentage ≤high school	34.1
Percentage white	98.0
Percentage with CHD/stroke/diabetes before 1999	33.3
Smoking in pack-years	21.8 (0–131)
Anxiety	0.2 (0–2.83)
Depression	0.2 (0–3.33)
Hostility	0.2 (0–3.00)
Happiness	7.4 (1–9)
Life satisfaction	7.9 (0–11)
Percentage basophils	0.6 (0–2)
Percentage eosinophils	3.2 (0–22)
Percentage lymphocytes	26.0 (5–90)
Percentage monocytes	8.8 (0–17)
Percentage neutrophils	61.65 (3–85)
Plasma folate (ng/mL)	17.41 (3.3–99.3)
DNA methylation in gene promoter regions (%)	
TLR-2	3.1 (0–8.9)
F3	2.3 (0–14.8)
NR3C1	47.0 (14.7–72.8)
ICAM-1	4.4 (1.7–16.1)
IFN-γ	84.4 (30.9–95.7)
IL-6	43.7 (10.3–86.6)
iNOS	69.7 (24.5–87.2)

CHD, coronary heart disease; F3, coagulation factor III; ICAM-1, intercellular adhesion molecule-1; IFN-γ, interferon-γ; IL-6, interleukin 6; iNOS, inducible nitric oxide synthase; NR3C1, glucocorticoid receptor; TLR-2, toll-like receptor 2.

Anxiety, depression and hostility scale scores were significantly positively correlated with one another, and were nearly all significantly inversely correlated with happiness and life satisfaction scores (all │r│>0.3 and p<0.01; table 2). By contrast, none of the methylation outcomes were moderately to strongly correlated with one another (all │r│<0.3; data not shown), suggesting that these outcomes represented relatively independent events and processes.

**Table 2:** Pearson correlation coefficients between psychological factors*

	Anxiety	Depression	Hostility	Happiness	Life satisfaction
Anxiety	1.00	0.76 (n=611)	0.67 (n=611)	−0.32 (n=612)	−0.31 (n=578)
Depression		1.00	0.63 (n=609)	−0.46 (n=611)	−0.42 (n=577)
Hostility			1.00	−0.30 (n=610)	−0.28 (n=577)
Happiness				1.00	0.58 (n=598)
Life satisfaction					1.00

*For men with observations for the pair of psychological factors.

p<0.01 for all correlations.

### Associations between psychological factors and average DNA methylation

Table 3 shows the multivariate-adjusted coefficient estimates from repeated measures models. Negative psychological factors were related to higher average methylation in *ICAM-1* promoter regions (with the associations for anxiety significant at the 0.10 level and for depression significant at the 0.05 level). Happiness was significantly inversely associated with *ICAM-1* promoter methylation. Depression was significantly positively associated, and happiness and life satisfaction were significantly inversely associated, with average methylation in *F3* promoter regions, respectively. For *TLR-2* promoter methylation, all negative psychological factors showed positive relations (with the association for hostility significant at the 0.10 level), and both positive psychological factors showed inverse relations (with the association for life satisfaction significant at the 0.05 level). For *iNOS* promoter methylation, all negative psychological factors showed inverse relations and both positive psychological factors showed positive relations. However, only the association for life satisfaction was significant at the 0.10 level. For *NR3C1* promoter methylation, depression, hostility, happiness and life satisfaction, all exhibited positive and non-significant associations. Likewise, psychological factors were inconsistently and non-significantly related to higher methylation in the promoter regions for *IFN-γ* and *IL-6*.

**Table 3:** Coefficient estimates (95% CI) for multivariate associations between psychological factors and average methylation in gene promoter regions, from repeated measures models

Gene							
	*TLR-2*	*F3*	*NR3C1*	*ICAM-1*	*IFN-γ*	*IL-6*	*iNOS*
Anxiety	0.07	0.17	−0.42	0.34^b^	0.50	0.36	−0.82
	(−0.17 to 0.32)	(−0.05 to 0.40)	(−1.54 to 0.71)	(−0.03 to 0.72)	(−0.41 to 1.40)	(−1.75 to 2.47)	(−2.28 to 0.64)
	n=558; 833 obs	n=607; 909 obs	n=581; 924 obs	n=548; 831 obs	n=640; 1069 obs	n=636; 1077 obs	n=499; 729 obs
Depression	0.08	0.34^a^	0.22	0.38^a^	0.21	−0.12	−0.60
	(−0.15 to 0.30)	(0.14 to 0.55)	(−0.76 to 1.21)	(0.04 to 0.72)	(−0.62 to 1.04)	(−2.07 to 1.83)	(−1.93 to 0.73)
	n=554; 825 obs	n=605; 904 obs	n=579; 919 obs	n=546; 826 obs	n=638; 1064 obs	n=634; 1071 obs	n=496; 723 obs
Hostility	0.22^b^	0.18	0.20	0.20	0.39	−0.54	−0.34
	(−0.04 to 0.49)	(−0.06 to 0.42)	(−1.00 to 1.40)	(−0.19 to 0.60)	(−0.56 to 1.34)	(−2.74 to 1.66)	(−1.82 to 1.14)
	n=554, 828 obs	n=603; 905 obs	n=578; 921 obs	n=545; 828 obs	n=636; 1066 obs	n=632; 1074 obs	n=497; 727 obs
Happiness	−0.02	−0.10^a^	0.12	−0.10^a^	0.04	−0.38	0.07
	(−0.09 to 0.05)	(−0.16 to −0.04)	(−0.17 to 0.41)	(−0.22 to −0.003)	(−0.20 to 0.28)	(−0.95 to 0.19)	(−0.33 to 0.47)
	n=582; 867 obs	n=636; 952 obs	n=608; 967 obs	n=577; 871 obs	n=669; 1117 obs	n=666; 1128 obs	n=523; 760 obs
Life satisfaction	−0.05^a^	−0.06^a^	0.09	−0.02	−0.04	0.15	0.20^b^
	(−0.09 to −0.01)	(−0.10 to −0.03)	(−0.09 to 0.26)	(−0.08 to 0.04)	(−0.19 to 0.10)	(−0.18 to 0.49)	(−0.02 to 0.43)
	n=539; 808 obs	n=590; 885 obs	n=563; 895 obs	n=538; 813 obs	n=619; 1036 obs	n=615; 1045 obs	n=481; 698 obs

Associations between each psychological factor and average levels of methylation across CpG sites within gene promoter regions examined in separate models. All models adjusted for age, smoking status, educational attainment, history of CHD or stroke prior to 1999, history of diabetes prior to 1999, % basophils, % eosinophils, % lymphocytes, % monocytes, % neutrophils and plasma folate.

^a^p<0.05.

^b^p^b^p<0.10.

CHD, coronary heart disease; TLR-2, toll-like receptor 2; F3, coagulation factor III; NR3C1, glucocorticoid receptor; ICAM-1, intercellular adhesion molecule-1; IFN-γ, interferon-γ; IL-6, interleukin 6; iNOS, inducible nitric oxide synthase.

For all associations significant at the 0.05 level, we further identified monotonic dose–response relationships, with categories of higher scores of the psychological factors being associated with stronger associations. Tables 4 and 5 show the coefficient estimates across categories as well as the p values from the tests for linear trend across categories; these p values were significant at the 0.05 level for *F3* promoter methylation and at the 0.10 level for *ICAM-1* promoter region methylation, respectively.

**Table 4:** Coefficient estimates from repeated measures models for multivariate associations between categorised scale values of depression, happiness, life satisfaction and *F3* promoter methylation (n=658 men, 988 observations)

	Coefficient estimate	95% CI	p Value
Depression			
0	–	–	–
0.01–0.4	−0.13	−0.34 to 0.09	0.24
>0.4	0.33	0.10 to 0.56	0.005
			P_trend_ =0.03
Happiness			
1–4 (unhappy)	–	–	
5–7	−0.20	−0.54 to 0.14	0.24
8–9 (happy)	−0.51	−0.85 to −0.18	0.003
			P_trend_ <0.001
Life satisfaction			
0–5	–	–	–
6–8	−0.28	−0.49 to −0.06	0.01
9–11	−0.40	−0.60 to −0.20	<0.001
			P_trend_ <0.001

*F3* methylation values corresponded to the average levels of methylation across CpG sites within the *F3* promoter region.

All models adjusted for age, smoking status, educational attainment, history of CHD or stroke prior to 1999, history of diabetes prior to 1999, % basophils, % eosinophils, % lymphocytes, % monocytes, % neutrophils and plasma folate.

CHD, coronary heart disease; F3, coagulation factor III.

**Table 5:** Coefficient estimates from repeated measures models for multivariate associations between categorised scale values of depression and happiness, and *ICAM-1* promoter methylation (n=600 men, 906 observations)

	Coefficient estimate	95% CI	p Value
Depression			
0	–	–	–
0.01–0.4	0.19	−0.16 to 0.55	0.29
>0.4	0.30	−0.09 to 0.70	0.13
			P_trend_ =0.09
Happiness			
1–4 (not happy)	–	–	–
5–7	−0.21	−0.76 to 0.34	0.46
8–9 (happy)	−0.42	−0.97 to 0.13	0.13
			P_trend_ =0.06

*ICAM-1* methylation values corresponded to the average levels of methylation across CpG sites within the *ICAM-1* promoter region.

All models adjusted for age, smoking status, educational attainment, history of CHD or stroke prior to 1999, history of diabetes prior to 1999, % basophils, % eosinophils, % lymphocytes, % monocytes, % neutrophils and plasma folate.

CHD, coronary heart disease; ICAM-1, intercellular adhesion molecule-1.

In all models, pack-years of smoking significantly predicted higher average methylation levels in the gene-specific promoter regions. Age was non-significantly inversely associated with methylation. Additional adjustment for household income (with lower income being non-significantly positively associated with methylation), baseline hypertension and total serum cholesterol, did not alter the main results (data not shown).

### Associations between psychological factors and serum ICAM-1

No psychological factors were associated with serum ICAM-1 levels (for anxiety: β=5.11, p=0.51; other psychological factors exhibited similar associations). ICAM-1 methylation levels and serum ICAM-1 levels were uncorrelated (r=−0.04).

## Discussion

In this study of community-dwelling elderly adult men, we found consistent associations between both, positive and negative psychological factors, with higher average leucocyte DNA methylation in *ICAM-1* promoter regions and in *F3* promoter regions. There was some evidence that hostility was positively associated with *TLR-2* promoter methylation, and that life satisfaction was inversely associated with both, *TLR-2* and *iNOS* promoter methylation. We observed less consistent and significant associations between psychological factors and average methylation for promoters of the genes for *NR3C1*, *IFN-γ* and *IL-6*.

Our main findings were generally robust across multiple BSI component scales. While this may stem from similarities across component scale measures, results using very different scales (eg, life satisfaction) were qualitatively consistent. Moreover, smoking has been linked to proinflammatory states and atherosclerosis,^45^ and the direction of the associations for smoking with hypermethylation of *ICAM-1* promoter regions matched those for negative psychological factors, providing support that the associations were not simply attributable to chance. Our findings were, furthermore, robust to the adjustment of the presence of CHD, stroke and diabetes, countering underlying comorbidities/health selection as alternative explanations for the main findings.

Higher circulating levels of serum *ICAM-1* have been previously independently linked to modest risks of CHD after adjusting for key covariates such as SES.^4^ ^46^ ^47^ Notably, we found no association between psychological factors and serum *ICAM-1*. Along with the presence of associations between psychological factors and *ICAM-1* promoter methylation, this could be explained by the fact that serum *ICAM-1* is derived from multiple sources (vascular endothelium, macrophages, lymphocytes), consistent with the absence of a correlation between leucocyte *ICAM-1* methylation and serum *ICAM-1*. Past investigations of the NAS have likewise found no association between serum *ICAM-1* and *LINE-1* leucocyte methylation levels.^48^ Whether methylation of *ICAM-1* in white cell count predicts serum *ICAM-1* levels derived solely from white cell count (vs other sources), and whether this *ICAM-1* independently contributes to higher risks of CHD, should be explored in future studies.

Atherosclerosis is a chronic inflammatory process involving the infiltration of leucocytes into the extravascular space, mediated in part by adhesion molecules. Smooth muscle cells participate in this process by expressing adhesion molecules such as *VCAM-1* and *ICAM-1*.^49^ *ICAM-1* plays a pivotal role in the adhesion of leucocytes to the endothelium.^50–52^ Given evidence that psychological factors are risk factors for atherosclerosis,^1^ one possible explanation for negative psychological factors being linked to higher *ICAM-1* promoter region methylation in leucocytes is *cellular signalling*, with *ICAM-1* being known to function via signal transduction^53^ ^54^ Low binding of leucocyte *ICAM-1* to its cell membrane integrins could trigger a cascade of proinflammatory mediators and signal endothelial cells to release *ICAM-1*,^52^ ^55–57^ and could thereby stimulate *ICAM-1* leucocyte binding to vascular endothelial cells. Hence, through signalling mechanisms, low leucocyte *ICAM-1* levels could induce leucocyte migration into vascular endothelial tissues. Future biological studies (eg, animal experiments that manipulate distress or other exposures) should further investigate and test this and other potential pathways.

Depression was positively associated, and happiness and life satisfaction were each inversely associated, with higher *F3* promoter methylation in leucocytes (which in turn would be linked to reduced leucocyte F3 expression). Some evidence suggests that the major source of *F3* in arterial thrombosis is the vascular wall rather than monocytes,^25^ although monocyte *F3* also contributes to inflammation and thrombosis. *F3*, also known as Tissue Factor, has been shown to be involved in cellular signalling and inflammatory pathways.^26^ ^27^ Similar to the hypothesis for *ICAM-1*, low leucocyte *F3* levels via signalling pathways may promote inflammatory states through greater vascular *F3* levels.

Furthermore, hostility was positively associated and life satisfaction was inversely associated with higher *TLR-2* promoter methylation, which would imply lower *TLR-2* expression. These findings appear contrary to the hypothesised role that *TLR-2* plays in atherosclerosis.^28^ ^29^ Nonetheless, there is some evidence to suggest that *TLR-2* promoter hypermethylation is present in chronic inflammatory processes such as periodontitis.^30^ In addition, it has been suggested that the inflammatory process itself may induce cytosine damage and aberrant methylation patterns, including hypermethylation.^58^ Furthermore, the association of negative psychological states such as hostility with decreased expression of TLR-2 may signify suppression of the immune system; this is consistent with observed relationships between stress and immune suppression in other studies.^59^

We found no associations between psychological factors and leucocyte *NR3C1* promoter methylation. Previous studies in humans have yielded conflicting results. For example, an investigation in prenatal women, using clinically-administered (Hamilton Rating) scales of anxiety and depression and a self-administered (Edinburgh Postnatal Depression) scale of depression, observed associations between higher maternal anxiety, and depressive symptom scores and methylation of CpGs within the promoter and exon 1F of the *NR3C1* gene (homologous to the l_7_ region of the rat *NR3C1* gene) in maternal blood leucocytes.^12^ A study of men and women aged 18–59 years reported correlations between a history of childhood adversity with higher leucocyte *NR3C1* gene promoter methylation, yet found no correlations for anxiety (using the State-Trait Anxiety Inventory) and only limited correlations for depression (using the Inventory for Depressive Symptoms) with GR promoter methylation (at 0 of 13 CpG sites and 2 of 13 CpG sites, respectively).^20^ Meanwhile, a recent brain postmortem study in adults found no hippocampal GR promoter methylation differences between those clinically diagnosed with major depression versus controls.^60^

Strengths of our study include its examination of multiple psychological factors (both positive and negative) and its novel exploration of DNA methylation in promoter regions of multiple genes plausibly involved in chronic immune/inflammatory processes and inflammation-related endothelial dysfunction; and its reliance on a community-based sample, which strengthens the generalisability of our findings. We further tested for and confirmed linear dose–response relationships, which supports the presence of causal associations.

There were several limitations to our study. First, we examined DNA methylation at a subset of CpG sites within a gene promoter region. The inability to assay high proportions, given methodological limitations, could have led us to the omission of some relevant CpG sites. The analysed CpGs (selected based on the aforementioned methodological limitations) may not necessarily have been good proxies for the rest of the CpGs within the same regions. Second, differences in results from previous studies, particularly for *NR3C1* methylation, might also stem from the measurement of methylation in peripheral blood rather than hippocampal tissue; methylation effects may be tissue-specific.^20^ ^61^ Third, due to the multiple associations examined, the multiple comparisons problem, whereby multiple comparisons may increase the presence of significant associations by chance, cannot be ruled out. Fourth, while the null associations for methylation in promoter regions of several genes including *NR3C1*, *IFN-γ* and *IL-6*, could reflect the true absence of associations, they could also possibly be attributed to selection bias due to attrition or missing methylation data, as suggested by demographic (age, education) differences in those analysed versus the NAS cohort in 1985, when the BSI was first administered. For instance, those with a stronger association between the psychological factors and methylation may have either died or been lost to follow-up, leading to attenuated and null associations in the analysed data. With respect to the varying sample sizes between analytic samples for genes examined, the mechanism of missing data due to insufficient DNA and assay failures was plausibly missing completely at random (MCAR), and entirely unrelated to the levels of methylation of a particular sequence of DNA.^32^ Under the MCAR mechanism, the listwise deletion method that we applied should be valid.^62^ In support of the MCAR assumption being met, we determined that those participants with and without missing methylation data for each gene were generally comparable on demographic characteristics (mean age, distribution of education), mean pack-years of smoking, and mean anxiety and depression scores. Fifth, the NAS cohort does not currently have genome-wide association study (GWAS) data. Hence, we could not specifically evaluate the interplay between genetics and DNA methylation and further studies are warranted. Sixth, we lacked measures of additional cell subtypes (eg, B cells, T cells and natural killer cells, as subtypes of lymphocytes), which may have biased our results through residual confounding. Finally, the presence of null associations may in part be due to the study sample being limited to an elderly, primarily white male population. Effects of psychological factors on DNA methylation may be more salient in other population subgroups, or at earlier, sensitive time points over the life course. Future studies should extend examination of these associations to younger adults, older women and members of other racial/ethnic groups.

In summary, our study primarily suggests novel relations between positive and negative psychological factors, and methylation of ICAM-1 promoter regions and linkages with F3 gene methylation, and, to a lesser extent, associations with *TLR-2* promoter methylation. Confirming these findings in other populations and settings may yield a better understanding of the epigenetic mechanisms by which psychological factors influence CHD and other major chronic disease outcomes.
